# Supplementary material for: Impact of Insulin Therapies on Cancer Incidence in Type 1 and Type 2 Diabetes: A Population-Based Cohort Study in Reggio Emilia, Italy
Source: Cancers (Basel). 2022 May 31;14(11):2719. doi: 10.3390/cancers14112719 (PMC9179836; doi:10.3390/cancers14112719)
Supplement: Supplementary file 1 [file cancers-14-02719-s001.zip › cancers-1731526-supplementary.pdf]

Supplementary materials

Supplementary Table S1. ATC codes of glucose lowering drugs considered in exposure assessment.

ATC code	Name	Category for exposure assessment	Chemical/pharmacological/therapeutic subgroups
A10AB01	insulin (human)	Insulin	Insulins and analogues for injection, fast-acting
A10AB04	insulin lispro	Insulin	Insulins and analogues for injection, fast-acting
A10AB05	insulin aspart	Insulin	Insulins and analogues for injection, fast-acting
A10AB06	insulin glulisine	Insulin	Insulins and analogues for injection, fast-acting
A10AC01	insulin (human)	Insulin	Insulins and analogues for injection, intermediate-acting
A10AC04	insulin lispro	Insulin	Insulins and analogues for injection, intermediate-acting
A10AD01	insulin (human)	Insulin	Insulins and analogues for injection, intermediate- or long-acting combined with fast-acting
A10AD04	insulin lispro	Insulin	Insulins and analogues for injection, intermediate- or long-acting combined with fast-acting
A10AD05	insulin aspart	Insulin	Insulins and analogues for injection, intermediate- or long-acting combined with fast-acting
A10AE01	insulin (human)	Insulin	Insulins and analogues for injection, long-acting
A10AE04	insulin lispro	Insulin	Insulins and analogues for injection, long-acting
A10AE05	insulin aspart	Insulin	Insulins and analogues for injection, long-acting
A10BA02	metformin	Met	Biguanides
A10BB01	glibenclamide	Sulf	Sulfonylureas
A10BB02	chlorpropamide	Sulf	Sulfonylureas
A10BB07	glipizide	Sulf	Sulfonylureas
A10BB08	gliquidone	Sulf	Sulfonylureas
A10BB09	gliclazide	Sulf	Sulfonylureas
A10BB12	glimepiride	Sulf	Sulfonylureas
A10BD01	phenformin and sulfonylureas	Met+Sulf	Combination (Biguanides + Sulfonylureas)
A10BD02	metformin and sulfonylureas	Met+Sulf	Combination (Biguanides + Sulfonylureas)
A10BD03	metformin and rosiglitazone	Met+OHA	Combination (Biguanides + Thiazolidinediones)
A10BD04	glimepiride and rosiglitazone	Sulf+OHAs	Combination (Sulfonylureas + Thiazolidinediones)

Supplementary Material

ATC code	Name	Category for exposure assessment	Chemical/pharmacological/therapeutic subgroups
A10BD05	metformin and pioglitazone	Met+OHAs	Combination (Biguanides + Thiazolidinediones)
A10BD06	glimepiride and pioglitazone	Met+OHAs	Combination (Sulfonylureas + Thiazolidinediones)
A10BD07	metformin and sitagliptin	Met+OHAs	Combination (Biguanides + Dipeptidyl peptidase 4 (DPP-4) inhibitors)
A10BD08	metformin and vildagliptin	Met+OHAs	Combination (Biguanides + Dipeptidyl peptidase 4 (DPP-4) inhibitors)
A10BF01	acarbose	OHAs	Alpha glucosidase inhibitors
A10BG02	rosiglitazone	OHAs	Thiazolidinediones
A10BG03	pioglitazone	OHAs	Thiazolidinediones
A10BH01	sitagliptin	OHAs	Dipeptidyl peptidase 4 (DPP-4) inhibitors
A10BH02	vildagliptin	OHAs	Dipeptidyl peptidase 4 (DPP-4) inhibitors
A10BH03	saxagliptin	OHAs	Dipeptidyl peptidase 4 (DPP-4) inhibitors
A10BX02	repaglinide	Sulf	Meglitinides (glinides)
A10BJ01	exenatide	OHAs	Glucagon-like peptide-1 (GLP-1) analogues
A10BJ02	liragludite	OHAs	Glucagon-like peptide-1 (GLP-1) analogues

Supplementary Table S2. ICD – 10 and ICD-O 3 codes for classification of cancer sites and morphology.

Supplementary Table S3. Diabetes duration in patients with type 2 diabetes by drug consumption, mean and standard error (SE) and ANOVA test for difference between groups the three groups of drug consumption and t-test for difference between the two groups of insulin users.

Group	mean (years)	SE	ANOVA	t-test
2.1 Insulin alone or in any combination (N=2282)	13.26	0.27	P<0.0001	P=0.0157
2.3 Insulin alone (N=1332)	14.16	0.24		
2.4 Untreated (diet-only) (N=4077)	3.39	0.08		

Supplementary Table S4. Number, incidence rates ratios (IRR) and 95% confidence intervals of cancer in other sites by type of treatment with type 1 and type 2 diabetes (DM1; DM2), aged 20-84 years

	Other sites							
	patients with DM2 in diet-only as reference				population without DM as reference			
	N	IRR	95%CI		N	IRR	95%CI	
Population with no DM (N=379,103)					3616	1.00	-	-
Population with DM (N=21,948)					378	1.09	0.98	1.22
1. Type 1 diabetes (N=758)					2	0.31	0.08	1.24
2. Type 2 diabetes (N=21,190)					376	1.10	0.99	1.23
2.1 Insulin alone or in any combination (N=2282)	32	0.79	0.47	1.33	32	0.90	0.64	1.28
2.2 Insulin alone (N=1332)	22	0.97	0.53	1.75	22	1.04	0.68	1.59
2.3 Untreated (diet-only) (N=4077)	78	1.00	-	-	78	1.17	0.94	1.47

Supplementary Table S5 – Number of neuroendocrine by site and morphology. No neuro endocrine cancers have been found in people with Type 1 DM.

Site and morphological group	no DM	DM2	Totale
Well differentiated not functioning endocrine carcinoma of pancreas and digestive tract	37	7	44
Well differentiated functioning endocrine carcinoma of pancreas and digestive tract	3	0	3
Poorly differentiated endocrine carcinoma of pancreas and digestive tract	73	4	77
Mixed endocrine-exocrine carcinoma of pancreas and digestive tract	0	0	0
Endocrine carcinoma of thyroid gland	14	2	16
Neuroendocrine carcinoma of skin	0	0	0
Typical and atypical carcinoid of the lung	26	3	29
Neuroendocrine carcinoma of other sites	224	41	265
Pheochromocytoma, malignant	3	2	5
Paraganglioma	1	0	1
**Total**	**381**	**59**	**440**

Code slection according to RARECARE (van der Zwan JM, Trama A, Otter R, Larrañaga N, Tavilla A, Marcos-Gragera R, Dei Tos AP, Baudin E, Poston G, Links T; RARECARE WG. Rare neuroendocrine tumours: results of the surveillance of rare cancers in Europe project. Eur J Cancer. 2013 Jul;49(11):2565-78) [29]. We selected all well, moderately and poorly differentiated, functioning and not functioning, gastroenteropancreatic neuroendocrine tumors, as well as thoracic and other sites neuroendocrine tumors (eg, lung, skin, thyroid, breast, etc).

Supplementary Table S6 – Number of neuroendocrine carcinoma of sites different by pancreas and digestive tract by type of diabetes

Neuroendocrine carcinoma of other sites

site	no DM	DM2	total
parotid	1		1
lung	190	37	227
mediastinum	1		1
breast	4		4
prostate	3		3
kidney	1		1
Bladder	11	2	13
endocrine glands	1		1
abdomen	2		2
lymph nodes	1		1
not specified	9	2	11
**total**	**224**	**41**	**265**

Supplementary Material

Site and morphology code selection for neuroendocrine neoplasm

Supplementary Table S7

*Well differentiated not functioning endocrine carcinoma of pancreas and digestive tract

gen neuro=1 if (icdo3t>="C150" & icdo3t<="C269") & (substr(morfo,1,4)=="8150" | substr(morfo,1,4)=="8240" | substr(morfo,1,4)=="8241" | substr(morfo,1,4)=="8242" | substr(morfo,1,4)=="8243" | substr(morfo,1,4)=="8244" | substr(morfo,1,4)=="8245" | substr(morfo,1,4)=="8248" | substr(morfo,1,4)=="8249")

replace neuro=2 if (icdo3t>="C480" & icdo3t<="C488") & (substr(morfo,1,4)=="8150" | substr(morfo,1,4)=="8240" | substr(morfo,1,4)=="8241" | substr(morfo,1,4)=="8242" | substr(morfo,1,4)=="8243" | substr(morfo,1,4)=="8244" | substr(morfo,1,4)=="8245" | substr(morfo,1,4)=="8248" | substr(morfo,1,4)=="8249")

*Well differentiated functioning endocrine carcinoma of pancreas and digestive tract

replace neuro=3 if (icdo3t>="C150" & icdo3t<="C269") & (substr(morfo,1,4)=="8151" | substr(morfo,1,4)=="8152" | substr(morfo,1,4)=="8153" | substr(morfo,1,4)=="8155" | substr(morfo,1,4)=="8156" | substr(morfo,1,4)=="8157")

replace neuro=4 if (icdo3t>="C480" & icdo3t<="C488") & (substr(morfo,1,4)=="8151" | substr(morfo,1,4)=="8152" | substr(morfo,1,4)=="8153" | substr(morfo,1,4)=="8155" | substr(morfo,1,4)=="8156" | substr(morfo,1,4)=="8157")

*Poorly differentiated endocrine carcinoma of pancreas and digestive tract

replace neuro=5 if (icdo3t>="C150" & icdo3t<="C269") & (substr(morfo,1,4)=="8002" | substr(morfo,1,4)=="8013" | substr(morfo,1,4)=="8041" | substr(morfo,1,4)=="8042" | substr(morfo,1,4)=="8043" | substr(morfo,1,4)=="8044" | substr(morfo,1,4)=="8045" | substr(morfo,1,4)=="8246")

replace neuro=6 if (icdo3t>="C480" & icdo3t<="C488") & (substr(morfo,1,4)=="8002" | substr(morfo,1,4)=="8013" | substr(morfo,1,4)=="8041" | substr(morfo,1,4)=="8042" | substr(morfo,1,4)=="8043" | substr(morfo,1,4)=="8044" | substr(morfo,1,4)=="8045" | substr(morfo,1,4)=="8246")

Supplementary Material

*Mixed endocrine-exocrine carcinoma of pancreas and digestive tract

```
replace neuro=7 if (icdo3t>="C150" & icdo3t<="C269") & (substr(morfo,1,4)=="8154")
```

*Endocrine carcinoma of thyroid gland

```
replace neuro=8 if icdo3t=="C739" & (substr(morfo,1,4)=="8002" |  substr(morfo,1,4)=="8013" | substr(morfo,1,4)=="8041" | substr(morfo,1,4)=="8240" | substr(morfo,1,4)=="8246" | substr(morfo,1,4)=="8249" | substr(morfo,1,4)=="8345" | substr(morfo,1,4)=="8346" | substr(morfo,1,4)=="8347" | substr(morfo,1,4)=="8510" |  substr(morfo,1,4)=="8512")
```

*Neuroendocrine carcinoma of skin

```
replace neuro=9 if (icdo3t>="C440" & icdo3t<="C449") & (substr(morfo,1,4)=="8002" |  substr(morfo,1,4)=="8013" | substr(morfo,1,4)=="8041" | substr(morfo,1,4)=="8240" | substr(morfo,1,4)=="8246" )

replace neuro=10 if (icdo3t>="C510" & icdo3t<="C519") & (substr(morfo,1,4)=="8002" |  substr(morfo,1,4)=="8013" | substr(morfo,1,4)=="8041" | substr(morfo,1,4)=="8240" | substr(morfo,1,4)=="8246" )

replace neuro=11 if icdo3t=="C600" & (substr(morfo,1,4)=="8002" |  substr(morfo,1,4)=="8013" | substr(morfo,1,4)=="8041" | substr(morfo,1,4)=="8240" | substr(morfo,1,4)=="8246" )

replace neuro=12 if icdo3t=="C609" & (substr(morfo,1,4)=="8002" |  substr(morfo,1,4)=="8013" | substr(morfo,1,4)=="8041" | substr(morfo,1,4)=="8240" | substr(morfo,1,4)=="8246" )

replace neuro=12 if icdo3t=="C632" & (substr(morfo,1,4)=="8002" |  substr(morfo,1,4)=="8013" | substr(morfo,1,4)=="8041" | substr(morfo,1,4)=="8240" | substr(morfo,1,4)=="8246" )

replace neuro=13 if substr(morfo,1,4)=="8247"
```

Supplementary Material

*Typical and atypical carcinoid of the lung

```
replace neuro=14 if substr(icdo3t,1,3)=="C34" & ( substr(morfo,1,4)=="8240" | substr(morfo,1,4)=="8241" | substr(morfo,1,4)=="8242" | substr(morfo,1,4)=="8243" |  substr(morfo,1,4)=="8244" | substr(morfo,1,4)=="8245" | substr(morfo,1,4)=="8249")
```

*Neuroendocrine carcinoma of other sites

```
replace neuro=15 if neuro==. & (substr(morfo,1,4)=="8002" |  substr(morfo,1,4)=="8013" | substr(morfo,1,4)=="8041" | substr(morfo,1,4)=="8042" | substr(morfo,1,4)=="8043" | substr(morfo,1,4)=="8044" | substr(morfo,1,4)=="8045" | substr(morfo,1,4)=="8150" | substr(morfo,1,4)=="8151" | substr(morfo,1,4)=="8152" | substr(morfo,1,4)=="8153"  | substr(morfo,1,4)=="8155" | substr(morfo,1,4)=="8156" | substr(morfo,1,4)=="8157" | substr(morfo,1,4)=="8240" | substr(morfo,1,4)=="8241" | substr(morfo,1,4)=="8242" | substr(morfo,1,4)=="8243" | substr(morfo,1,4)=="8244" | substr(morfo,1,4)=="8245" | substr(morfo,1,4)=="8246" | substr(morfo,1,4)=="8248" | substr(morfo,1,4)=="8249")
```

*Pheochromocytoma, malignant

```
replace neuro=16 if (icdo3t>="C740" & icdo3t<="C749") & (substr(morfo,1,4)=="8700")
```

*Paraganglioma

```
replace neuro=17 if substr(morfo,1,4)=="8680" | substr(morfo,1,4)=="8693"
```
